# Glycosylated hemoglobin A1c as a marker predicting the severity of coronary artery disease and early outcome in patients with stable angina

**DOI:** 10.1186/1476-511X-13-89

**Published:** 2014-05-29

**Authors:** Li-Feng Hong, Xiao-Lin Li, Yuan-Lin Guo, Song-Hui Luo, Cheng-Gang Zhu, Ping Qing, Rui-Xia Xu, Na-Qiong Wu, Jian-Jun Li

**Affiliations:** 1 Division of Dyslipidemia, State Key Laboratory of Cardiovascular Disease, Fu Wai Hospital, National Center for Cardiovascular Diseases, Chinese Academy of Medical Sciences, Peking Union Medical College, Beijing 100037, China; 2 Divison of Cardiology, Guangci Hospital affiliated Medical College of Wuhan University & the Fifth Hospital of Wuhan, Wuhan 430050, China

**Keywords:** Hemoglobin A1c, Stable angina pectoris, Coronary artery disease, Outcome

## Abstract

**Background:**

Glycosylated hemoglobin A1C (HbA1c) has been widely recognized as a marker for predicting the severity of diabetes mellitus (DM) and several cardiovascular diseases. However, whether HbA1c could predict the severity and clinical outcomes in patients with stable coronary artery disease (CAD) remains largely unknown. We determine relationship of HbA1c with severity and outcome in patients with stable CAD.

**Methods:**

We enrolled 1433 patients with stable angina who underwent coronary angiography and were followed up for an average 12 months. The patients were classified into three groups by tertiles of baseline HbA1c level (low group <5.7%, n = 483; intermediate group 5.7 - 6.3%, n = 512; high group >6.3%, n = 438). The relationships between the plasma HbA1c and severity of CAD and early clinical outcomes were evaluated.

**Results:**

High HbA1c was associated with three-vessel disease. Area under the receivers operating characteristic curve (AUC = 0.67, 95% CI: 0.63-0.71, P < 0.001) and multivariate logistic regression analysis suggested that HbA1C was an independent predictor of severity of CAD (OR = 1.60, 95% CI: 1.29-1.99, P < 0.001) even after adjusting for gender, age, risk factor of CAD, lipid profile and fasting blood glucose. During follow-up, 133 patients underwent pre-specified outcomes. After adjusting for multiple variables in the Cox regression model, HbA1C remained to be an independent predictor of poor prognosis (HR = 1.28, 95% CI: 1.12-1.45, P < 0.001).

**Conclusions:**

We concluded that high level of baseline HbA1c appeared to be an independent predictor for the severity of CAD and poor outcome in patients with stable CAD.

## Introduction

Glycosylated Hemoglobin A1c (HbA1c) is a pivotal biomarker reflecting both fasting and postprandial plasma glucose concentration over the preceding 2–3 months [1,2]. And also, it has been regarded as an important tool in diabetes diagnosis and management [1-5]. It has been reported that elevated HbA1c levels probably mean long-term insulin resistance and severe consequences such as hyperglycemia, dyslipidemia, hypercoagulability, and system inflammatory response [5-7]. Furthermore, several studies have demonstrated a positive relationship between elevated HbA1c and poor outcome in the setting of general population, acute coronary syndrome (ACS), acute myocardial infarction (AMI), heart failure, pancreatitis, and even patients after coronary artery bypass surgery and drug-eluting stent (DES) implantation with and without primary diabetes mellitus (DM) [8-17]. Although consistent evidences have supported that optimal control of HbA1c at a target value can confer to a lower incidence of microvascular complications both in type 1 and type 2 DM, the association of high levels of HbA1c with macrovascular disease such as stable coronary disease remains controversial [11,12,18-21].

Moreover, a few population studies suggested that the values of plasma HbA1c should be perceived as a continuous variable without a normal cut-off point because it might exhibit a significantly detrimental role even when it was in “normal” or relatively low levels [6,7]. More interestingly, it has also been showed that a higher level of circulating HbA1c was related to elevated inflammatory markers such as C-reactive protein (CRP), fibrinogen and white blood cell count, which were routinely available and well established predictors of future mortality. Therefore, it might provide meaningfully predictive value than either alone [6,7]. Meanwhile, several previous studies have demonstrated a positive correlation of high HbA1c levels with severity of coronary artery disease while the clinical significance of previous observation were apparently limited by either a retrospective design manner or small sample size [22-26]. According to the best of our knowledge, data from larger sample in a prospective design regarding the value of plasma HbA1c in predicting the disease severity in patients with stable coronary artery disease (CAD) is not currently available. Furthermore, whether the elevated HbA1c levels could also provide any additional prognostic information for those patients with stable CAD remains to be determined.

The aim of this study, therefore, was to prospectively investigate the predictive power of HbA1c levels for the severity and the early outcome in patients with stable CAD.

## Materials and methods

### Study population

This was a single center, prospective follow-up study. From June 2011 through March 2012, we prospectively enrolled 1433 consecutive women and men (70.3%) aged 19 to 86 years (average age 58.29 years) with typical stable exertional angina pectoris referred for selective coronary angiography at our center. Patients with ACS, significant hematologic disorders (white cell count <3.5×10^9^/L or >20×10^9^/L), infectious or inflammatory disease, severe liver and/or renal insufficiency were excluded from the present study. Data including the demographic, clinical, laboratory findings and angiographic examination were collected from all patients.

The protocol of this study was approved by Fu Wai hospital ethics committee, and complied with the Declaration of Helsinki. The informed consent was obtained from all participants.

### Diagnostic criteria

Hypertension was defined by SBP ≥ 140 mmHg and/or DBP ≥ 90 mmHg, or currently receiving antihypertensive medication [27]. The diagnostic criteria recommended by WHO for diabetes mellitus were used [28]. According to these criteria, we diagnosed patients as having diabetes with fasting blood glucose values ≥126 mg/dl, or 2-h blood glucose ≥200 mg/dl, or under the active treatment with insulin or oral hypoglycemic agents. Hyperlipidemia was considered to be present in patients with fasting total cholesterol (TC) ≥200 mg/dl or triglyceride (TG) ≥150 mg/dl. CAD was defined as the presence of significant obstructive stenosis at least 50% of the vessel lumen diameters in any of the main coronary arteries by at least two independent senior interventional cardiologists based on quantity coronary angiography. The severity of CAD was scored as 0 (absent or minimal atherosclerotic involvement), 1 (single vessel disease), 2 (two-vessel disease), 3 (three-vessel disease and/or left main stem disease and/or equally affected of left anterior descending and left circumflex branch). The left ventricular ejection fraction was evaluated by echocardiograph using the area-length methods or modified Simpson’s rule.

### Biochemical examination

Venous blood samples were obtained from each patient at baseline on admission. HbA1c levels were measured using the Tosoh G7 Automate HPLC Analyzer (TOSOH Bioscience, Japan). The concentrations of high-sensitivity C-reactive protein (hs-CRP) were determined using immunoturbidimetry (Beckmann Assay 360, Bera, Calif., USA). TC and TG were measured by enzymatic methods and high-density lipoprotein cholesterol by a direct method (Roche Diagnostics, Basel, Switzerland). Low-density lipoprotein cholesterol was obtained by Friedewald’s formula (if fasting triglycerides <3.39 mmol/l) or by ultracentrifugation. Apolipoprotein B was measured by an immune-turbidimetric method (Tina-quant, Roche Diagnostics) calibrated against the World Health Organization/International Federation of Clinical Chemistry reference standard SP3–07. All other included biomarkers were analyzed by standard hematological and biochemical tests.

### Follow up

All patients were subjected to follow up throughout the study period with an average of 12 months by a telephone and/or clinical interview. The pre-specified clinical end points were defined as cardiac death, nonfatal MI, revascularization, and re-hospitalization due to attack of acute coronary syndrome (ACS).

### Statistical analysis

Continuous variables and categorical variables were analyzed by the chi-squared statistic tests, the one-way analysis of variance test, or the Kruskal-Wallis test when appropriate. Receivers operating characteristic (ROC) curves were constructed at the most discriminating cutoff point values to predict CAD. The relationship of HbA1c with angiographic findings were evaluated by univariate and multivariate logistic regression models (including potential factors such as age, gender, cardiac risk factors, medicine treatment, DES implantation, baseline lipid profile and other hematological index) using forward stepwise selection process. The association of HbA1c with 12-month outcomes was determined with Cox proportional hazard models using forward stepwise selection process. Event-free survival curves were constructed using the Kaplan-Meier methods and compared using log-rank test. All analyses were performed using SPSS version19.0 software (Chicago, Illinois, USA).

## Results

### Baseline characteristics

**Figure 1:**
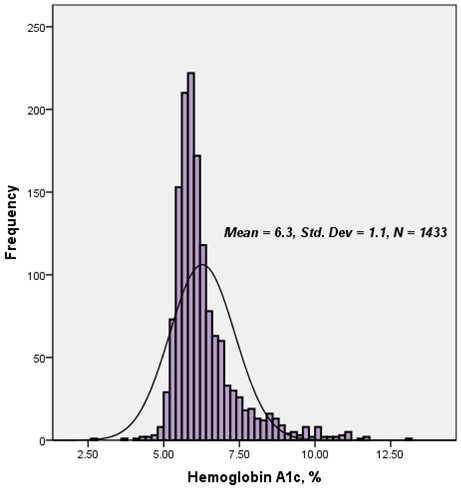
Distribution of baseline serum hemoglobin A1C level in the study population.

The baseline demographic, clinical characteristics and laboratory findings according to tertiles of HbA1c (low group <5.7%, n = 483; intermediate group 5.7-6.3%, n = 512; high group >6.3%, n = 438) were shown in Table 1. The distribution of HbA1c was shown in Figure 1. As shown in Table 1, patients with the higher HbA1c levels were more likely to be older, female, and have elevated body mass index (BMI) and a prior history of hypertension and dyslipidemia. Meanwhile, the pattern of the inflammation-related biomarkers such as hs-CRP, leucocyte count, fibrinogen and D-dimer were significantly unbalanced among the patients. Additionally, patients with the higher levels of HbA1c received the more drug administrations and DES implantations.

**Table 1:** Baseline characteristics according to the tertiles of serum hemoglobin A1C levels

**Variables**	**Tertiles of serum hemoglobin A1C levels (%)**				**P-value**
	**Total (n = 1433)**	**Low (<5.7; n = 483)**	**Intermediate (5.7 ~ 6.3; n = 512)**	**High (>6.3; n = 438)**	
Demographic					
Age, years	58.3 ± 10.2	55.5 ± 10.8	59.6 ± 9.6	59.8 ± 9.5	<0.001
Male gender	1008(69.9)	365(75.6)	350(68.3)	293(66.9)	0.008
Risk factors Body mass index (kg/m^2^)	25.5 ± 3.2	25.0 ± 3.0	25.4 ± 3.3	26.0 ± 3.2	<0.001
Current Smoking	770(53.4)	271(56.1)	272(53.1)	227(51.8)	0.404
Hypertension	909(63.4)	280(58.0)	317(61.9)	312(71.2)	<0.001
Diabetes mellitus	374(26.1)	89(18.4)	93(18.2)	192(43.8)	<0.001
Hyperlipidemia	1064(74.2)	306(63.4)	393(76.8)	365(83.3)	<0.001
Peripheral vascular disease	26(1.8)	6(1.2)	11(2.1)	9(2.1)	0.509
Prior Stroke	68(4.7)	16(3.3)	27(5.3)	25(5.7)	0.182
Family history of CAD	158(11.0)	44(9.1)	55(10.7)	59(13.5)	0.104
Laboratory date					
LVEF (%)	62.2 ± 8.1	62.5 ± 8.2	62.1 ± 8.2	62.2 ± 7.9	0.672
NT-pro-BNP (fmol/mL)	710.4 ± 515.7	663.9 ± 422.7	720.8 ± 492.3	749.5 ± 622.1	0.036
FBG (mmol/L)	5.5 ± 1.5	6.8 ± 2.1	5.0 ± 0.6	4.7 ± 0.6	<0.001
Haemoglobin (g/L)	139.9 ± 15.1	141.5 ± 14.9	139.7 ± 14.7	138.3 ± 15.9	0.006
Leucocyte count (10^9^/L)	6.4 ± 1.6	6.2 ± 1.6	6.4 ± 1.6	6.6 ± 1.7	0.002
Platelet count (109/L)	205.1 ± 60.7	201.8 ± 68.6	208.4 ± 58.6	204.9 ± 53.7	0.221
Fibrinogen (g/L)	3.2 ± 0.8	2.9 ± 0.6	3.2 ± 0.7	3.3 ± 0.9	<0.001
D-dimer (mg/dL)	0.4 ± 0.7	0.3 ± 0.4	0.4 ± 0.6	0.3 ± 1.0	0.023
hs-CRP (mg/L)	2.9 ± 3.6	2.1 ± 2.9	3.0 ± 3.5	3.6 ± 4.1	<0.001
Endothelin-1 (fmol/mL)	0.6 ± 0.3	0.6 ± 0.3	0.6 ± 0.3	0.6 ± 0.3	0.492
Albumin (g/L)	41.8 ± 3.5	41.6 ± 3.3	41.9 ± 3.8	41.8 ± 3.4	0.491
Cr (umol/L)	75.6 ± 16.2	75.4 ± 13.8	75.4 ± 17.1	76.2 ± 17.5	0.686
UA (mmol/L)	347.4 ± 4.2	354.0 ± 83.3	348.8 ± 80.5	338.5 ± 88.8	0.017
Lipid profile					
Triglycerides (mmol/L)	4.2 ± 1.1	4.1 ± 0.9	4.2 ± 1.1	4.1 ± 1.1	0.008
Total cholesterol (mmol/L)	4.2 ± 1.1	4.0 ± 0.9	4.2 ± 1.1	4.2 ± 1.1	0.077
LDL-C (mmol/L)	2.5 ± 0.9	2.5 ± 0.9	2.5 ± 0.8	2.5 ± 0.9	0.497
HDL-C (mmol/L)	1.1 ± 0.3	1.1 ± 0.3	1.1 ± 0.2	1.1 ± 0.3	0.051
Lipoprotein (a) (mg/L)	236.9 ± 244.6	220.6 ± 224.7	265.6 ± 263.4	221.4 ± 240.4	0.004
ApoA (g/L)	1.5 ± 0.3	1.5 ± 0.3	1.5 ± 0.2	1.5 ± 0.3	0.920
ApoB (g/L)	1.1 ± 0.3	1.0 ± 0.3	1.1 ± 0.3	1.1 ± 0.3	0.157
Medical treatment					
Aspirin	1358(94.8)	444(91.9)	490(95.7)	424(96.8)	0.002
Clopidogrel	1297(90.5)	416(86.1)	473(92.4)	408(93.2)	<0.001
Beta-blocker	1129(78.8)	351(72.7)	415(81.1)	363(82.9)	<0.001
ACE-I	424(29.6)	132(27.3)	162(31.6)	130(29.7)	0.389
Statin	1332(93.0)	429(88.8)	486(94.9)	417(95.2)	<0.001
DES implantation	250(17.4)	60(12.4)	109(21.3)	81(18.5)	0.001

CAD = Coronary artery disease; LV-FE = Left ventricular ejection fraction; hs-CRP = high sensitivity C-reactive protein; LDL-C = Low density lipoprotein cholesterol; HDL-C = High density lipoprotein cholesterol; ACE-I = Angiotensin converting enzyme inhibitors; DES = Drug eluting stent.

### HbA1c for predicting the extent of CAD

**Figure 2:**
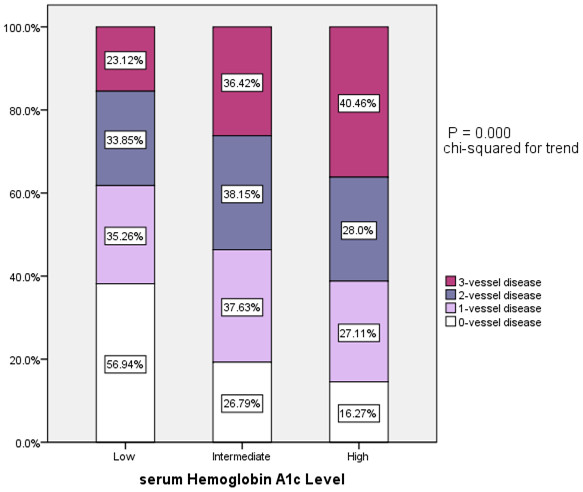
Association of tertiles of serum hemoglobin A1C levels and extent of coronary artery disease.

**Figure 3:**
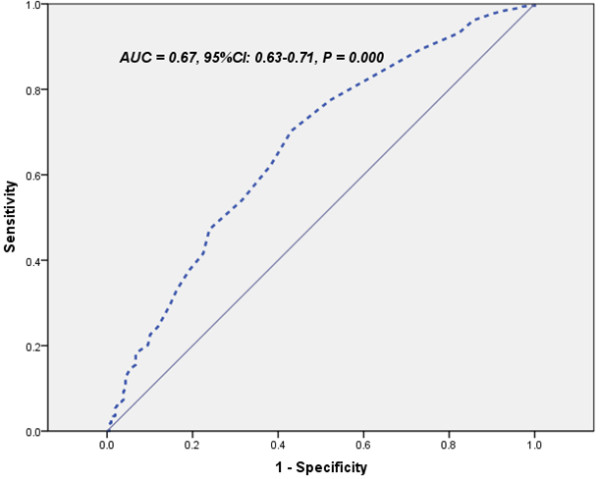
ROC curves showed discriminatory power of serum hemoglobin A1C levels on coronary artery disease.

There were a statistically significant correlation between the tertiles of plasma HbA1C and angiographic characteristics (chi-squared for trend, P <0.001, Figure 2). The multivariate logistic regression analysis showed that the plasma HbA1C level was an independent predictor of the presence of CAD after adjusting for conventional risk factors of CAD (OR = 1.60, 95% CI: 1.29-1.99, P <0.001, Table 2) . Area under the ROC curves (AUC) analysis also indicated the well discriminatory power of HbA1c levels for the presence of CAD (AUC = 0.67, 95% CI: 0.63-0.71, P <0.001, Figure 3).

**Table 2:** Unadjusted and adjusted predictive value of serum hemoglobin A1C levels for coronary artery disease

**Variables**	**Unadjusted**		**Adjusted**	
	**OR (95% CI)**	**P-value**	**OR (95% CI)**	**P-value**
Gender	3.06(2.00-4.68)	<0.001	3.34(2.41-4.64)	<0.001
Age	1.04(1.03-1.06)	<0.001	1.04(1.02-1.06)	<0.001
Dyslipidemia	2.26(1.62-3.17)	<0.001	2.33(1.69-3.22)	<0.001
Hemoglobin A1c	1.76(1.32-2.34)	<0.001	1.60(1.29-1.99)	<0.001

### HbA1c for predicting the early outcomes

**Figure 4:**
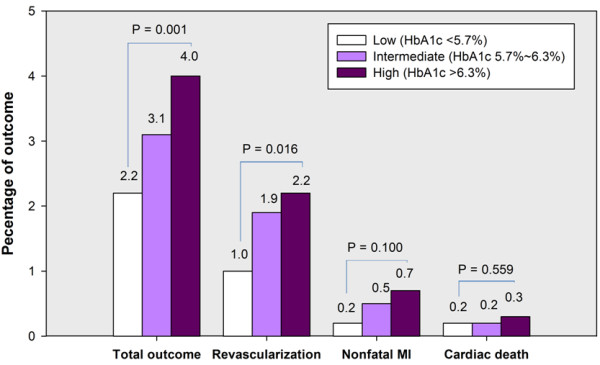
Association of tertiles of serum hemoglobin A1C levels and 12-month outcome.

**Figure 5:**
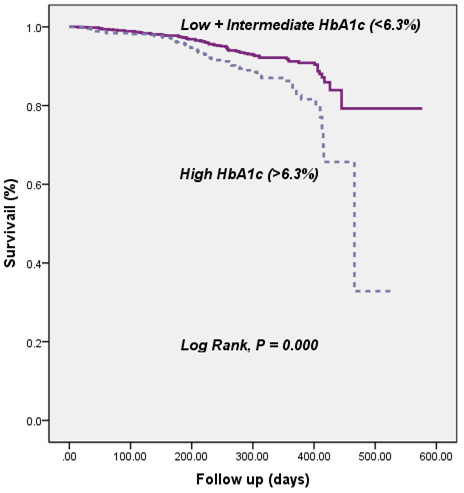
Kaplan-Meier curve for cumulative event-free survival based on tertiles of baseline plasma hemoglobin A1c levels.

The population of the present study was followed up for an average of 12 months follow-up (ranged from 14 to 577 days). During follow-up, 133 patients suffered from adverse outcome (Figure 4). There were a significant association between baseline plasma HbA1c levels and incidence of total outcome, revascularization (P = 0.001 and P = 0.016, respectively), but not for nonfatal MI or cardiac death (P = 0.100 and 0.559, respectively) during follow-up. The multivariate analysis showed that plasma HbA1C remained to be an independent predictors of overall outcome for patients with stable angina (HR = 1.28, 95% CI: 1.12-1.45, P <0.001) except for the family history of CAD and numbers of affected coronary arteries (Table 3) after adjusting for all potential confounders including age, gender, cardiovascular risk factors, medicine treatment, DES implantation, baseline lipid profile and other hematological index.Kaplan-Meier curves for cumulative event-free survival based on the tertiles of baseline HbA1c were presented in Figure 5. As presented in Figure 5, elevated plasma HbA1c (>6.3%) were generally associated with increased early adverse outcome (P < 0.001). However, subgroup analysis indicated the predictive power of plasma HbA1c was only found in patients with stable angina who had a history of DM (P < 0.001), but not in populations without a history of DM (P = 0.324).

**Table 3:** Unadjusted and adjusted predictive value of serum hemoglobin A1C levels for 12-month total outcome

**Variables**	**Unadjusted**		**Adjusted**	
	**HR (95% CI)**	**P-value**	**HR (95% CI)**	**P-value**
Family history of CAD	2.05(1.03-4.06)	0.040	1.99(1.01-3.93)	0.046
Numbers of affected coronary arteries	1.44(1.20-1.73)	<0.001	1.42(1.19-1.69)	<0.001
Hemoglobin A1C	1.29(1.07-1.57)	0.008	1.28(1.12-1.45)	<0.001

CAD = coronary artery disease.

## Discussion

The present study prospectively investigated the predictive value of plasma HbA1c for the severity of CAD and mortality in patients with stable CAD. Consistent with previous small sample size studies [24-26], our data also demonstrated that elevated HbA1C levels significantly conferred to clinical discriminators not only for the presence of CAD, but also for the severity of coronary lesions in patients with CAD. However, both chi-squared for trend and multivariate Cox proportional regression analysis with adjustment for all potential confounders in our study showed the usefulness of HbA1c in predicting the early outcome of the enrolled population. Kaplan-Meier curve for cumulative event-free survival based on the tertiles of baseline plasma HbA1c levels apparently showed that the elevated HbA1c levels (>6.3%) were generally associated with increased early adverse outcome. More interestingly, subgroup analysis indicated that the predictive power of plasma HbA1c existed only in patients with DM who presented as stable CAD but not in populations without the history of DM. The results clearly suggested the important role of baseline plasma HbA1c in diabetic patients with CAD.

It has been demonstrated that DM is a risk for the development of CAD and individuals who had DM will suffer from more intensive atherosclerotic lesions and more cardiovascular events [29,30]. Previous studies have already suggested an association of high-normal glucose and HbA1C level with the presence of CAD in a variety of individuals such as in patients with or without diabetes, in asymptomatic persons, even in general population with undiagnosed diabetes [3,22,24,31-33]. However, to the best of our knowledge, there is no data available regarding the role of HbA1C in the prognostic predictor of patients with DM. In this study, we prospectively enrolled a large cohort of patients who presented as typical angina-like chest pain (stable angina) to evaluating the relationship between HbA1C and CAD. We found that HbA1C could significantly confer to clinical discriminators not only for the presence of CAD but also for the severity of coronary lesions in these patients. The results analyzed by univariate and multivariate logistic regression models indicated that plasma HbA1C levels was an independent predictors of the presence of CAD after adjusting for other risk factors of CAD and lipid parameters. AUC evaluation suggested a well discriminatory power of HbA1c for CAD in our population studied.

Indeed, HbA1C has been proposed as reliable tool for not only diagnosing DM but also identifying individuals at high risk of cardiovascular events with and without DM [1,19]. Although accumulating evidence suggested that an elevated HbA1C level was clearly linked to a poor prognostic outcome in patients with AMI or ACS, even after cardiac surgery or coronary stent implantation, the prognostic value of baseline HbA1c in patients of stable angina has not been well established [11,19,20]. In the present study, we detected that the higher level of plasma HbA1c was relevant to adverse prognosis in patients with stable CAD during an average of 12 month follow-up. Among adverse cardiovascular events, the patients with higher HbA1c were more prone to receive the coronary intervention of revascularizations in agreement with previous studies. Therefore, the present study confirmed and extended previous studies regarding the role of HbA1c in predicting the severity and early outcome in stable CAD.

The underlying hypothesis of the current results might consisted in that the high levels of HbA1c were not only associated with the long-term disorder of glycolipid metabolism but also connected with low-grade systematic inflammation and atherosclerotic plaques progress [34]. Our data might supported this hypothesis because we found that higher levels of HbA1c in the population studied were clearly associated with the adverse baseline characteristics such as higher cardiovascular risk profile and higher inflammatory biomarkers such as hs-CRP, leukocyte counts, fibrinogen, D-dimer, uric acid and so on. Our data were also in agreement with previous evidence that suggested a correlation between plasma HbA1c with above inflammatory biomarkers, chemical parameters, either alone or combined. These markers or risk factors had a direct role on the progression of atherosclerotic artery disease and adverse cardiovascular events [7,31,35-39]. Moreover, previous studies also suggested that the higher plasma HbA1c might indicate a potential impact of hyperglycemia on the vasculature before the establishments of formal diagnosis for clinically DM and/or overt atherosclerosis disease [7,40]. Therefore, as a long half-life protein, HbA1c might be involved in both chronic inflammatory response and acute active phase of ACS, resulting in accelerating the progress and rupture of atherosclerotic lesions.

Summarily, in this prospective, a large cohort, short-term outcome study, the data clearly suggested that high level of plasma HbA1C (>6.3%) was an independent predictor for the presence and severity of CAD as well as the early outcome of patients with stable angina. Long-term follow-up may be needed for further revealing more information regarding the role of HbA1C in diabetic patients with stable CAD.

## Abbreviations

HbA1c: Hemoglobin A1C; CAD: Coronary artery disease; DM: Diabetes mellitus.

## Competing interests

The authors declare no conflicts of interests.

## Authors’ contributions

L-FH wrote the manuscript. J-JL conceived and designed the study, reviewed and edited of manuscript. L-FH and X-LL collected and analyzed the data. Y-LG, S-HL, C-GZ, PQ, R-XX and N-QW searched the literatures and collected data. All authors read and approved the final manuscript.
